# Ethics Education in COVID-19: Preclinical Medical Students’ Approach to Ventilator Allocation

**DOI:** 10.7759/cureus.16976

**Published:** 2021-08-07

**Authors:** Canon Brodar, Carly Muller, Kaitlyn E Brodar, Jeffrey P Brosco, Kenneth W Goodman

**Affiliations:** 1 Medicine, Miller School of Medicine, Miami, USA; 2 Psychology, University of Miami, Coral Gables, USA; 3 Psychology, Mailman Center for Child Development, Miami, USA; 4 Clinical Pediatrics, Miller School of Medicine, Miami, USA; 5 Clinical Pediatrics, Institute for Bioethics and Health Policy, Miami, USA; 6 Bioethics & Health Policy, Institute for Bioethics and Health Policy, Miami, USA

**Keywords:** medical education, innovation in medical education, medical ethics and pandemic, triage, bioethics, ventilator strategies

## Abstract

Introduction

COVID-19 has confronted clinicians with a potential need to ration ventilators. There is little guidance for training medical students to make such decisions in future practice. How students would make ventilator triage decisions remains unknown.

Methods

One hundred fifty-three medical students in 18 problem-based learning groups participated in a ventilator-rationing exercise in April 2020 as part of an ethics curriculum adapted in response to the COVID-19 pandemic. Students were provided with a prompt requiring fictional patients to be prioritized for ventilators in the face of scarce resources. The authors reviewed group responses, tallied triage criteria, and identified approaches to triage decisions.

Results

The most common triage criteria were patient comorbidities, clinical status, age/life stage, prognosis, life expectancy, and an individual’s role in pandemic response. Additional criteria included quality of life, ventilator availability, public perception, and patient need. Students approached triage decisions by developing systems for triage, appealing to empirical evidence and academic literature, making value judgments, and identifying adjuncts and alternatives to triage.

Discussion

With minimal input from educators, students learned key ethical principles in triage medicine, recapitulated approaches to triage described in the clinical and bioethics literature, and suggested methods for tolerating distress of complex ethical decisions. Medical education should equip students to critically consider bioethical concerns in triage and prepare for possible moral distress during public health crises.

## Introduction

Throughout the COVID-19 pandemic, health systems have grappled with the need to allocate resources in the face of real or potential scarcity. In response, institutions have developed and instituted crisis standards of care, underscoring the need for policies and ethical discourse guiding the rationing of health resources [1,2].

The need to ration resources is not new, but the problem of rationing ventilators is rare in well-resourced countries. Engaging in this form of triage presents a unique source of distress for clinicians [3]. Moral injury - which includes feeling that one’s actions or inactions (or witness of another’s actions or inactions) violate moral values [4] - has been linked to post-traumatic stress symptoms in physicians, and COVID-19 has exacerbated this issue [5,6]. This trauma underscores the need for bioethics in medical education to prepare students for challenging ethical decisions and to help develop resilience in the midst of moral uncertainty [7].

A systematic review of studies on medical student training in pandemic/disaster medicine suggests that the current methods and content of disaster-response training need to be adapted to better prepare students and their response to COVID-19 [8]. Though the Association of American Medical Colleges (AAMC) has advocated for medical student training in disaster medicine, a 2012 survey of AAMC-accredited schools found only 31% included disaster medicine in curricula, and only 10.3% taught principles of triage [9]. Prior studies of triage decision-making in medical education have focused on such disaster curricula or emergency-response skills [10,11]. Existing literature on medical education around resource allocation thus focuses on preparedness and management of mass casualty events rather than responses to large-scale public health disasters and lacks discussion of relevant bioethical considerations.

The COVID-19 pandemic demonstrates the need to prepare future clinicians for public health crises and reveals a gap in understanding how physicians and trainees approach resource allocation. This study seeks to address this gap in the literature by describing how preclinical medical students responded to a prompt regarding ventilator allocation during COVID-19 as part of a problem-based learning (PBL) exercise within an ethics curriculum. Authors initially developed and implemented the exercise for educational purposes rather than for research, but the outcomes may be beneficial for understanding clinician decision-making and developing future bioethics curricula. Analyses thus aimed to identify three key components. Aim one was to determine how preclinical medical students ranked patients with varied clinical presentations. Aim two was to describe students’ triage criteria. Aim three examined students’ approach to the problem of triage. 

## Materials and methods

Study design and setting

This study involved the implementation of a case-based learning exercise developed for educational purposes and further analysis of student responses. Participants were second-year students of the professionalism, ethics, and legal medicine (PELM) course (N=153) at the Miller School of Medicine - University of Miami. These students, self-organized into 18 groups of approximately eight students, had previously participated in PBL sessions throughout the academic year. Prior PELM topics included confidentiality, valid consent, and end-of-life decision-making. One series of cases focused on patient access to care, and in 2020, a COVID-19 case was added to highlight ethical issues in resource allocation. PBL groups were asked to teleconference for 30 to 40 minutes and write a consensus answer to the COVID-19 case. There were no related lectures or faculty input. However, students were required to read an essay on ventilator triage related to COVID-19 [3]. Answers were not required to be unanimous, and students were asked to keep track of dissenting opinions. Students submitted final responses for the course and participated in a large group review session to discuss responses.

Materials

The course instructor (JB) developed a case vignette that asked students to determine which patients with COVID-19 would be provided a ventilator or palliative care in the context of the increasing scarcity of healthcare resources. All names in the case were fictitious. See Table 1 for a description of each patient and Appendix A for the full vignette. Responses included in-line comments on patients and/or summary paragraphs of 65 to 889 words (mean=348). Responses were de-identified, and a standardized institutional self-certification tool determined that institutional review board approval was not required for this study.

**Table 1:** Summary of patient information from the case and student triage decisions MICU: Medical Intensive Care Unit; T10:  Tenth thoracic vertebra

Fictional Patient Name	Age	Sex or Gender	Occupation	History	No. Groups Allocating Ventilator (%)	Mean Priority Rank (Range)
Anselmo Aguilar	59	man	stock-broker	T-10 paraplegia, cancer, multi-organ failure from sepsis	0 (0%)	4.5 (4-5)
Maite Pennyman	25	woman	works at a grocery store	Down syndrome with no significant co-morbidity except for atrial septal defect repaired at birth	11 (78.6%)	1.8 (1-3)
Antonio Philippe Archetto	92	male	lawyer	Cardiac output <50%	0 (0%)	4.4 (4-5)
Dr. Aurelia Jimenez	45	woman	critical care physician	Infected while working in MICU	12 (85.7%)	1.2 (1-3)
Carlos Johnson	59	man	mayor of Miami-Dade County	History of hypertension, type 2 diabetes	7 (58.3%)	2.3 (1-3)

Data analysis

For aim one, we tabulated decisions for each patient in the vignette. Groups provided explicit treatment recommendations for individuals (i.e., ‘ventilator’ vs. ‘palliative care’ or 'no ventilator'; N=14), a rank-order for prioritizing patients for ventilator allocation (N=11), or both (N=7). Of groups providing explicit recommendations, five deferred decisions for one or more patients for whom they desired further information. We thus present the data in two ways: the frequency with which patients were given a ventilator (for groups giving explicit recommendations) and average priority ranking for receiving a ventilator. We calculated mean rankings from assigned consecutive numerical rankings based on the order in which groups prioritized patients (1=highest to 5=lowest). If two patients were prioritized equally, they were assigned the same rank, and the next consecutive rank was skipped (e.g. ranks of 1, 2, 3, 3, 5).

For aim two and three, we first independently reviewed the responses and noted key themes relevant to ethics. This process generated twelve codes related to triage criteria and four codes related to triage decisions.

CB and CM coded each response for triage criteria. We collaboratively discussed discrepancies in coding until we reached a consensus. We tallied the usage of triage criteria codes and examined how they were used across responses. Next, CB and CM coded each response for the way in which the group described their approach to triage decisions. We again collaboratively discussed the results, particularly in regard to implications for training in bioethics.

## Results

Students’ triage rankings

Fourteen groups provided explicit ventilator decisions (i.e., ventilator vs. palliative care or 'no ventilator') for some or all of the fictional patients in the triage case. Most allocated a ventilator for the youngest and healthiest patients: Aurelia Jimenez, a 45-year-old physician who was infected while working in the Medical Intensive Care Unit (N=12), and Maite Pennyman, a 25-year-old female grocery-store worker with Down syndrome (N=11). Half of the groups allocated a ventilator for Carlos Johnson, a 59-year-old mayor with a history of hypertension and type 2 diabetes (N=7). None allocated ventilators for Anselmo Aguilar, a 59-year-old male stockbroker with the greatest number of comorbid conditions (T10 paraplegia, cancer, and multi-organ failure from sepsis), or the oldest patient Antonio Archetto, a 92-year-old lawyer with a cardiac output <50%.

Eleven groups ranked patients for ventilator priority, and seven of these prioritized patients in addition to providing the aforementioned recommendations. Based on mean ranking across groups, Jimenez (1.2/5) and Pennyman (1.8/5) were highest ranked, followed by Johnson (2.3/5). Consistent with ventilator decisions, Archetto (4.4/5) and Aguilar (4.5/5) were ranked with the lowest priority to receive a ventilator (refer to abovelisted Table 1 for allocation decisions and mean priority rankings). 

Triage criteria

We identified 12 triage criteria used in ventilator allocation decisions. All groups based triage decisions on comorbidities (N=18). The second-most common criteria were age/life stage and clinical status, e.g. 'healthy' or 'decompensating already' (N=17). Prognosis or short-term survivability was used in 16 responses, e.g. 'good chance of survival' contributed to triage decisions. Longer-term life expectancy was used less commonly (N=13), and related comments generally referred to age as well.

Utility (N=15) and occupation/status (N=13) were the next most common criteria. Comments on utility focused on the ability of the physician and mayor to respond to the pandemic and on the essential work of the grocery-store employee. Descriptions of utility often overlapped with occupation/status that indicated a patient’s job or social status (e.g., being an 'active member of society') rather than a role in the pandemic response. Other criteria included futility (N=11), quality of life (N=8), ventilator availability (N=8), public perception (N=8), and need (N=3). While some groups discussed disability in their triage decisions, this was not an independent criterion, and considerations of disability in these responses were previously analyzed and reported separately [12]. See Table 2 for sample quotes of each identified criterion and the number of groups using the criteria for triage decisions. 

**Table 2:** Triage criteria and frequency of use for student triage decisions EF = Ejection Fraction

Criterion	No. of Groups Using Criterion ( % )	Sample Quotes
Comorbidities	18 (100.0%)	“We do not know of any other comorbidities … that would compromise her recovery...” “... pre-existing low EF would make it less likely that he could recover from COVID even if given ventilation.”
Age/Life Stage	17 (94.4%)	“Should get a ventilator because he has a high chance of survival with a ventilator due to age.”
Clinical Status	17 (94.4%)	“We recommend for this patient to receive ventilation since she is … presumably healthy.”
Prognosis	16 (88.9%)	“Good chance of survival, healthy.”
Utility	15 (83.3%)	“[Jimenez] could contribute significantly to society/healthcare if she is able to recover and come back to work.”
Life Expectancy	13 (72.2%)	“Given [Archetto’s] age and anticipated remaining years of life, he would likely be lower on the hierarchy in terms of ranking who gets the ventilator.”
Occupation/Status	13 (72.2%)	“[Jimenez] was sickened in course of duty –most of us agreed that we would give priority to health care workers.”
Futility	11 (61.1%)	“Given his condition, putting him on a ventilator would likely just prolong his suffering.”
Ventilator Availability	8 (44.4%)	“[Offer] palliative care if not enough ventilators.”
Public Perception	8 (44.4%)	“The loss of a leader could cause potential downstream effects such as policy on handling the situation and mass hysteria.” “Have to be careful about [this] being considered discrimination.”
Quality of Life	7 (38.9%)	“… he might have a better quality of life if treated with a ventilator than [Aguilar] ...”
Need	3 (16.7%)	“In reality, who presented first with the need for a ventilator would also be a large contributing factor...”

Not all decisions were unanimous, as some groups noted disagreement (N=13) or uncertainty (N=11), and some expressed concern for biases that may affect decisions (N=8). Responses with dissenting opinions noted disagreement over how to address factors such as quality of life, race, gender, disability, or whether to prioritize healthcare workers or political leaders. Some disagreements were due to difficulty deciding between patients depending on the number of ventilators available, which was not directly stated in the prompt. Groups that noted uncertainty about treatment decisions often expressed a desire for further clinical information. 

Recognizing the danger of bias in the context of triage, some groups specified details that were not used as the basis for their decisions: “We attempted to eliminate biases on opinions on the quality of life and contribution to society with our scoring system.” Others identified how bias might intersect with their decision: “status as the mayor of Miami-Dade - for better or worse - is likely a contributing factor in [Johnson] receiving a ventilator with higher priority than others.”

Approaches to triage decisions

We identified four primary ways students approached triage decisions: developing a system for triage, appealing to evidence or academic literature, making value judgments, and identifying adjuncts and alternatives to triage. 

System for Triage

The first approach to the ventilator allocation problem involved describing a system for triage (N=8). The simplest system involved some method where groups determined which criteria should be prioritized over others, or developed priority levels or scoring systems for patients based on some criteria (N=5):

We used the guiding principles that we should give [ventilators to] individuals who: (1) had a greater chance of improving on a ventilator (remembering that ventilators have their own risks), (2) are young and have not gone through all of life’s phases, (3) do not have complicated comorbidities that would reduce chances of survival, and (4) are health care providers or first responders contributing to health care in the pandemic.

One group described exclusion criteria for which patients should not receive ventilators: “Severe conditions would be disqualifying because these would make it very unlikely to survive.” Another group proposed a lottery system for patients with equivalent prognoses. While such descriptions varied in length and content, they indicated a principled or organized approach to the problem of triage and provided a level of objectivity and consistency to groups’ decisions on ventilator allocation.

Evidence and Academic Literature

Nearly half of the groups (N=7) appealed to empirical evidence or academic literature to make triage decisions. Four groups reported data such as mortality rates of patients with COVID-19, sepsis, organ failure, or advanced age to support decisions to withhold ventilators, e.g., “Aguilar has the serious comorbid condition of sepsis, which according to one review, increases the risk of mortality by 17-22% compared to 2% in non-septic patients before considering the risk of mortality associated with ventilation.” Students used peer-reviewed papers, preprints, and other academic sources to support their arguments by providing evidence for risks and benefits of ventilator use, methods for ventilator splitting, or reference to an academic medical center’s guidance on resource allocation, e.g., “We came up with these factors with the assistance of the bioethics criteria for resource allocation from the University of Washington.”

Value Judgments

Beyond stating facts about the case or weighing medical decisions about patient triage, many groups supported triage decisions with value judgments that indicate some form of moral or ethical position (N=12). While the content of these statements varied across groups, this category of statements indicated opinions on “right” or “wrong” aspects of triage.

For example, for groups that used occupation as a basis for triage, some explained their decisions by briefly stating an intention to promote the “greater good” or that they employed a “utilitarian perspective.” Some groups provided more detailed accounts of their deliberations. One group, in particular, supported the consideration of occupation or utility in prioritizing healthcare workers as a matter of justice:

Half of our group believed that healthcare workers should be given some priority, for several reasons. First, it ensures that healthcare workers are not disincentivized from coming to work given the high risk of contracting the virus. Second, it confirms "reciprocal justice": physicians knowingly work on the frontline of pandemics at great cost to their own health, so it would be unjust to not provide them with the best possible medical care when they fall sick doing this work. Third, they could be a significant resource to save more lives in the pandemic overall. If Dr. Jimenez made a full recovery, she’d be an invaluable resource during the pandemic as a fully trained ICU physician with antibodies against COVID. In this way, Dr. Jimenez is the best option if driven by the principle of "the most lives saved."

On the other hand, another group identified triage as an uncomfortable ethical dilemma and considered how prioritizing patients based on occupation - or at all - might be unjustly dependent on biases:

Clearly, this question is placing us into the ethical dilemma of how we value one human life as opposed to another. In this question, there seems to be enough medically justifiable reasons for choosing one participant over another. We could imagine a situation in which one could be placed in an uncomfortable position of choosing one patient over another without such medical reasons, a scenario in which many decisions might be based upon the typical biases of our society - ageism (92 y/o), ableism (Downs patient), elitism (mayor and critical care doctor). The most important takeaway is that there is actually no one variable that makes one more deserving of receiving life supporting care as all deserve this equally.

These statements indicate value judgments with explicit references to the moral beliefs or ethical principles underlying group decisions, i.e. that healthcare workers should be cared for, that triage decisions should save the most lives possible, or that all patients equally deserve access to life support.

Other statements revealed value judgments in describing their system for triage. One group noted the complexity, subjectivity, and potential resultant guilt of triage decision-making, and in response, they proposed the use of standard operating procedures (SOPs), a triage committee, and risk score calculations:

In this setting, it is more complicated than simply choosing who should and should not receive the ventilator. First, there needs to be a SOP to determine who would benefit most from and who would be granted the ventilator. It needs to be a uniform procedure that can be replicated in repeat settings. Secondly, there needs to be an SOP and COVID committee that bears the responsibility of deciding who gets the ventilator. The burden of guilt needs to be taken away from the physician or provider providing direct care to the patient. There needs to be an automatic calculation of risk scores for each individual patient, making the decision more objective. However, this is an impossible and very difficult decision to make as an individual.

In establishing a method to protect those involved in the ventilator allocation process, students evaluated triage as potentially hazardous to patients and clinicians, and thus implied a belief that both parties ought to be safeguarded. 

While some revealed value judgments in the creation of a system for triage, others did so by setting limits on triage. Two groups opposed the withdrawal of ventilators for re-allocation to other patients: “since Mr. Aguilar is already intubated, it would be unacceptable to simply take him off to re-allocate to another patient.” One group supported a first-come, first-served approach to triage, thus expressing opposition to withholding care:

In reality, who presented first with the need for a ventilator would also be a large contributing factor, as we would not do nothing for a patient under the justification that we were saving medical devices in case someone else came along who would either need it more or have a better chance of improving from such intervention.

In describing what a group would not do as a part of ventilator allocation, such statements demonstrate a proscriptive approach to triage that underlines a set of professional values.

Lastly, the simplest form of a value judgment included an expression of regret or lament, such that this was a “horrible situation,” or a wish to treat all patients equally, e.g. “Preferably, we would be able to ventilate any patient who would medically benefit from it, and who consents to it.” While such statements do not explicitly describe a moral or ethical opinion, they signal that the group has considered the problem of triage and assessed that the situation is not as it ought to be or desired that things would be different.

Adjuncts and Alternatives to Triage

Some groups took an indirect approach to triage decisions by describing adjuncts and alternatives to triage (N=5). Such responses provide a creative or subversive approach to the prompt by attempting to work around the prioritization of patients for ventilator allocation. 

First, some groups described a technological approach to the problem. One group suggested attempting alternative treatment options and ventilator splitting:

[We would] assess the degree of ventilator need in all of these patients, as there is new evidence that high flow nasal cannulas and proning has increased benefit over ventilator support in many COVID patients. We only need to have people in the hospital if they are hypoxic; otherwise encourage going home and self-isolation with monitoring of symptoms. Next, deferring to hospital policy on appropriate ventilator use is essential, though splitting the ventilators exclusively between COVID patients could be an option for the future, though this is controversial.

Another group similarly described the use of “medical judgment” to circumvent the need for triage, and together, these proposals attempt to alleviate the burden of decision-making by reducing the need for triage by applying medical knowledge and technology.

Other groups voiced a need for systems-change in response to scarcity, suggesting transfer to other hospitals, contacting governments for supplies, or a better preparation for future emergencies, e.g. “Precaution should be exhibited to be prepared for emergencies like this in future, and more ventilators should be ordered and stored in case of emergencies.” Some groups also discussed communication-based strategies for responding to the problem, including conversations on goals of care, the use of advanced directives and living wills, and early involvement of the palliative care team. Along with the use of technology, systems-change and communication strategies were suggested as alternatives to triage that would reduce the need for ventilator allocation or as adjuncts to triage that would ease its process.

## Discussion

To our knowledge, this is the first report describing medical student ventilator allocation decisions for a PBL exercise. Without explicit curricular content regarding these issues, student responses independently recapitulated criteria and approaches to triage identified in the bioethics literature and institutional crisis standards of care [13]. Triage decisions were based on the limited information provided within the exercise prompt but were driven most commonly by clinical characteristics, secondarily by utilitarian concerns for pandemic emergency response, and occasionally by other concerns. Age/life stage and life expectancy were common criteria across groups, which is consistent with the proposed basis of triage on short-term prognosis, long-term prognosis, and lifecycle considerations per the findings of a focus group study on community values regarding ventilator allocation [14].

For groups that provided more detailed responses to the triage prompt, answers explained or contextualized decisions beyond a binary yes/no ventilator allocation decision or priority ranking with some form of justification. Provision of such justification has itself been used as a measure of the effect of bioethics education [15]. In our case, it may also serve a protective purpose in the face of the “burden of guilt” that one group described. The development of a system for triage shifts the burden of decision-making from the individual to a process based on clinical and ethical principles, thereby safeguarding both patients and clinicians from dangers of triage such as those identified in student responses, e.g., unfair individual biases and the moral distress of making “impossible” decisions.

The other approaches to triage we observed may serve a similar purpose. An appeal to the academic literature provides reassurance that triage decisions are thoughtfully considered and well-founded. Statements of values signal good intentions and acknowledge the dangers of triage. Seeking adjuncts and alternatives to triage demonstrates an effort to maximize the use of available resources and suggests discomfort with triage. Overall, student responses provided rationales for triage decisions that demonstrate reasoned approaches to bioethical problems and reveal an inclination to make ethical treatment decisions and prevent moral injury.

This educational exercise allowed students to independently engage with triage issues and communicate on contentious topics with their peers and faculty, thus simulating bioethical challenges faced during the COVID-19 pandemic. In the past, educational exercises have been designed to prepare medical trainees for pandemics with knowledge and understanding of healthcare systems and essential public health resources [16,17]. Given the heterogeneity of health systems, competencies for pandemic response may be variable and dependent on local resources. Bioethics should be incorporated into such training to provide students with flexible tools to make and communicate ethical decisions in future crises.

As a preliminary study, our work is limited in that the educational exercise was not initially developed for research. Further, responses were consensus answers, and individual views regarding ventilator allocation were not assessed. Students involved in the exercise were in the preclinical portion of their education and should be assessed after exposure to the clinical environment. Our results are from a single institution and may not be generalizable to settings with different curricular approaches. Despite these limitations and given the dearth of literature on the subject, our results may be valuable for understanding student approaches to ethical problems and for designing bioethics curricula for medical trainees.

Given that the study was not designed to investigate quantitative results of student triage, it is important to note that our triage results are not generalizable to patient outcomes. Triage decisions may be based upon and biased by numerous factors including fictional patient names and occupations, and this level of complexity is intended to simulate the real-life challenge of triage. Future research and educational exercises could limit variables from patient history to study or address specific biases.

Based on the results of our study, we propose that goals of education on triage should be two-fold: (1) to equip students to incorporate both medical knowledge and bioethical consideration into patient-management decisions and (2) to prepare students for the moral distress associated with difficult decisions. Educators who include triage in medical school curricula should therefore adjust constraints on the limits of resources available in the exercise to limit adjuncts and alternatives to decision-making and thereby encourage engagement with bioethical issues while also ensuring opportunities for debriefing.

## Conclusions

In the context of a case-based learning exercise, we found medical students made group decisions for ventilator allocation based on criteria similar to those proposed in the bioethics literature and most commonly included patient comorbidities, clinical status, age/life stage, prognosis, life expectancy, and an individual’s role in the pandemic response. Student approaches to triage included describing systems for triage, appealing to empirical evidence and academic literature, describing value judgments, and identifying adjuncts and alternatives to triage. These approaches may indicate how students tolerate the uncertainty of complex ethical decisions.

Medical education should use such cases to equip students to critically consider bioethical problems and prepare them for possible moral distress in future public health crises. Future research should evaluate the beliefs and attitudes of clinicians regarding ventilator allocation and the potential role of problem-based learning in bioethics in promoting moral resilience in healthcare students.

## Appendices

Appendix A

Professionalism, Ethics and Legal Medicine (PELM) Cases for Small Groups & Tutor Guide

Access to Health Care Covid-19 Case: Crisis Standards of Care

Mr. Aguilar was one of many patients who needed a ventilator that day.  After his diagnosis of cancer, he lapsed into multi-organ failure from sepsis and required immediate intubation and ventilator support.  At the same time, dozens of patients infected with Covid-19 came to the hospital as the pandemic surge hit Miami. As hospitals across the county scrambled to find additional resources, physicians realized that there were only a few ventilators and health care teams left for the many patients who needed to be intubated.

Among the following patients, to whom would you choose to provide palliative care, and to whom would you choose to provide a ventilator?  Why?  What reasons can you provide for your choice?

Mr. Anselmo Aguilar, a 59-year-old man with T-10 paraplegia who works as a stock-broker, and now has multi-organ failure from sepsis in addition to his underlying diagnosis. 

Maite Pennyman, a 25-year-old woman with Down syndrome who works at Publix.  No significant comorbidity except for an atrial septal defect repaired at birth.

Antonio Philippe Archetto, a 92-year-old male who still practices law and had a cardiac output before Covid-19 infection of less than 50%.

Dr. Aurelia Jimenez, a 45-year-old critical care physician infected with Covid-19 while working in the medical ICU.

Carlos Johnson, the 59-year-old mayor of Miami-Dade County with a history of hypertension and type 2 diabetes.
